# Post-Infarction Left Ventricular Aneurysm Repair

**DOI:** 10.21470/1678-9741-2023-0449

**Published:** 2025-02-11

**Authors:** Ugur kaya, Alperen Yildiz, Abdurrahim Colak

**Affiliations:** 1 Department of Cardiovascular Surgery, Faculty of Medicine, Ataturk University, Erzurum, Turkey. E-mail: dr.ugurum@hotmail.com; 1 Department of Cardiovascular Surgery, Faculty of Medicine, Ataturk University, Erzurum, Turkey; 1 Department of Cardiovascular Surgery, Faculty of Medicine, Ataturk University, Erzurum, Turkey

Dear Editor,

We read the letter of Evora et al.^[1]^ about our article entitled “Application of circular patch plasty (Dor procedure) or linear repair techniques in the treatment of left ventricular aneurysms”^[2]^. We thank them for their evaluations.

In the years when technical modifications were discussed, Jatene et al.^[3]^ stated that the deterioration in the integrity of normal spiral muscle bands with aneurysm formation occurs in both transfer and longitudinal directions, and they emphasized the advantages of septum plication after scar tissue resection, circular reduction in the aneurysm orifice, and repair of the orifice using a patch when necessary. They reported that lower mortality and better ventricular functions were achieved with this method. Based on this concept, Evora et al.^[4]^ stated that their variant technique can be used as an easily applicable and safe method for the repair of left ventricular aneurysms.

Although it seems technically significant at a theoretical level in terms of both improving ventricular functions and reducing mortality, the lack of large prospective randomized series showing the difference between the techniques has caused the classical linear repair and endoventricular circular patch plasty methods to continue to be two of the most used techniques in many clinics. Kessler et al.^[5]^ stated that they did not find any significant difference between linear repair and circular repair techniques in terms of both anatomical and ventricular functions in 62 patients.

On the other hand, it is suggested that the targeted improvement in ventricular functions is achieved at the highest level with the endoventricular technique in which circular repair is performed, but it has not been fully demonstrated in later studies. Most likely, the more precise application of three-dimensional echocardiography, computerized tomography and magnetic resonance techniques will help to understand the small differences between the techniques in the postoperative period^[6]^.

In this study, we wanted to state that these two surgical methods can be applied with low mortality and morbidity if sufficient ventricular cavity is provided by publishing our results of surgery performed with the classical linear technique and endoventricular circular patch plasty (Dor) using the suturing technique to partially provide longitudinal shortening. According to our repair results, we believe that linear repair or endoventricular circular patch plasty techniques can be used with acceptable surgical risks until the differences are clearly clarified with more detailed studies^[2]^.
